# Performance of a SARS CoV-2 antibody ELISA based on simultaneous measurement of antibodies against the viral nucleoprotein and receptor-binding domain

**DOI:** 10.1007/s10096-021-04284-5

**Published:** 2021-06-04

**Authors:** Nina Reiners, Carolin Schnurra, Henning Trawinski, Judith Kannenberg, Thomas Hermsdorf, Andrea Aebischer, Torsten Schöneberg, Sven Reiche, Christian Jassoy

**Affiliations:** 1 Institute for Medical Microbiology and Virology, Leipzig University Hospital and Medical Faculty, University of Leipzig, Johannisallee 30, 04103 Leipzig, Germany; 2 Division of Infectious Diseases and Tropical Medicine, Department of Medicine II, Leipzig University Hospital, Leipzig, Germany; 3 Rudolf Schönheimer Institute of Biochemistry, Medical Faculty, University of Leipzig, 04103 Leipzig, Germany; 4 Department of Experimental Animal Facilities and Biorisk Management, Friedrich Loeffler Institute, Federal Research Institute for Animal Health, Greifswald, Insel Riems, Germany

**Keywords:** SARS CoV-2, Nucleoprotein, Receptor-binding domain, COVID-19, Antibodies, ELISA

## Abstract

SARS CoV-2 antibody assays measure antibodies against the viral nucleoprotein (NP) or spike protein. The study examined if testing of antibodies against both antigens increases the diagnostic sensitivity. Sera (N=98) from infected individuals were tested with ELISAs based on the NP, receptor-binding domain (RBD), or both proteins. The AUROCs were 0.958 (NP), 0.991 (RBD), and 0.992 (NP/RBD). The RBD- and NP/RBD-based ELISAs showed better performance than the NP-based assay. Simultaneous testing for antibodies against NP and RBD increased the number of true and false positives. If maximum diagnostic sensitivity is required, the NP/RBD-based ELISA is preferable. Otherwise, the RBD-based ELISA is sufficient.

**Supplementary Information:**

The online version contains supplementary material available at 10.1007/s10096-021-04284-5.

## Introduction

Infection with the SARS CoV-2 induces a virus-specific immune response [1–4]. IgG antibodies against the virus indicate previous infection and examination of the antibody response in a population provides information about the level of exposure to the virus [5–8].

Commercial SARS CoV-2 antibody tests are based on measuring antibodies against the viral nucleoprotein (NP); the spike glycoprotein regions S1, S2, or the receptor-binding domain (RBD); or, more rarely, a combination of two viral proteins [9–15]. Antibody tests based on either the NP or the spike protein have similar sensitivity but react with slightly different sets of sera [16–19]. This suggests that tests that concurrently measure antibodies against the two proteins are more sensitive than tests for antibodies against only NP or the spike glycoprotein. Based on this assumption, the aim of the study was to examine if simultaneous testing for IgG against the NP and RBD recognizes more sera than testing for antibodies against NP or RBD alone.

## Study design

We performed a diagnostic study to compare the diagnostic sensitivity and specificity of IgG antibody ELISAs based on SARS CoV-2 NP, RBD, or the two proteins simultaneously.

### Serum samples

Serum (N=91) and plasma (N=89) collected before 2019 were tested as putative negative specimens. Plasma and 80 sera were from blood donors (Institute for Transfusion Medicine, University Clinics, University of Leipzig). Eleven sera were from laboratory personnel. Ninety-eight sera were from adults with a positive SARS CoV-2 RT-PCR and mild to moderate or no symptoms. Forty-eight of the sera were obtained 4–10 weeks after infection; 50 blood specimens were drawn 6 months after infection. Participants were infected in March or April 2020. The sera from 4–10 weeks have previously been used to compare the sensitivity of commercial antibody tests [18]. Most (46 of 50) of the sera were from the same individuals.

### Viral antigens

The SARS CoV-2 nucleoprotein was produced in *Escherichia coli* as a maltose-binding domain fusion protein. The receptor-binding domain (RBD) was generated as a strep-tag fusion protein in Expi293 cells (see Suppl. Material S1).

### Antibody ELISA

Antibody ELISAs were performed with antigen-coated microtiter plates according to a modified standard protocol (see Suppl. Material S2).

### Calibrator and cut-off value

A SARS CoV-2 antibody–positive serum prediluted in a protein stabilizer (Antibody Stabilizer PBS, Candor Bioscience GmbH) was used as the calibrator sample. To establish the calibration factor, the calibrator and 91 control sera from before 2019 were tested in parallel on the same plate. The calibration factor was calculated as the ratio of the OD of the calibrator serum and the mean plus two standard deviations of the OD of the control sera. In subsequent assays with plasma and sera, the cut-off was calculated by dividing the OD of the calibrator sample by the calibration factor.

### Data analysis

Measurement units (U) were calculated by dividing the OD of the serum sample by the cut-off OD. As a start, sera with less than 1.0 U were negative and sera higher than 1.0 were positive. Receiver operating characteristic (ROC) analysis was performed and the cut-off values of the tests were adjusted such that the ELISAs had similar specificity. Sensitivity and specificity of the ELISAs were calculated with a contingency table and ROCs were calculated with a spreadsheet program. The area under the receiver operating characteristic curve (AUROC) was calculated with the MedCalc statistical software and the AUROCs were compared with VassarStats (http://vassarstats.net/index.html). The 95% confidence intervals were calculated using the MedCalc “Test for one proportion” and the Clopper-Pearson confidence interval. Proportions of unpaired serum samples were compared with the MedCalc “Comparison of proportions calculator” that uses the chi-squared test (https://www.medcalc.org/calc/comparison_of_proportions.php). Proportions of paired serum samples were compared with the exact binomial test (https://scistatcalc.blogspot.com/2013/11/mcnemars-test-calculator.html).

## Results

### Area under the receiver operating characteristic curve (AUROC)

Receiver operating characteristic analysis was performed to determine the AUROC as a measure for the discrimination level of the tests. The AUROCs of the NP-, RBD-, and NP/RBD-based test were 0.958 (95% confidence interval (CI): 0.927–0.978), 0.991 (95% CI: 0.971–0.998), and 0.992 (95% CI: 0.974–0.999), respectively. The AUROC of the RBD- and NP/RBD-based assays was significantly larger than the AUROC of the NP-based ELISA (p-values 0.039 and 0.032, Table 1).

**Table 1:** Area under the receiver operating characteristic curve (AUROC) of the SARS CoV-2 antibody ELISAs

	NP	RBD	NP/RBD
AUROC	0.958 CI: [0.927; 0.978]	0.991* CI: [0.971; 0.998]	0.992* CI: [0.974; 0.999]

*p-values of two-tailed comparisons of the AUROC of the antibody assays. NP- and RBD-based ELISA: 0.039, NP- versus NP/RBD-based ELISA: 0.032

### Comparison of the sensitivity of the assays

To compare the diagnostic sensitivity of the tests, the cut-offs of the ELISAs were adjusted to a specificity of 98.33% by ROC analysis. The cut-off values were taken to determine the sensitivity of the tests with sera from SARS CoV-2-infected individuals. The NP-based ELISA found antibodies in 81 of the sera (82.7%, 95% confidence interval (CI): 73.7–89.6%). The RBD-based test showed 91 positive results (92.9%, 95% CI: 85.9–97.1%) and the NP/RBD-based assay recognized 94 (95.9%, 95% CI: 89.9–98.9%) of the sera. Subgroup analysis with the 48 sera obtained 4–10 weeks after infection showed positivity rates of 95.8% (95% CI: 85.7–99.5%), 97.9% (95% CI: 88.9–99.95%), and 100% (95% CI: 92.6–100%). The 50 sera from 6 months after infection gave 70.0% (95% CI: 55.4–82.1%), 88.0% (95% CI: 75.7–95.5%), and 92.0% (95% CI: 80.8–97.8%) positive results. The diagnostic sensitivity of the NP-based and NP/RBD-based tests was lower with sera from 6 months than from early after infection (Table 2).

**Table 2:** Sensitivity of the nucleoprotein (NP), RBD-based, and NP/RBD-based IgG ELISAs at a specificity of 98.3%

		Positive sera (%)		
Time post infection	No. of sera	NP	RBD	NP/RBD
> 4 weeks	98	82.7 CI [73.74; 89.60]	92.9 CI [85.89; 97.11]	95.9 CI [89.85; 98.87]
4–10 weeks	48	95.8 CI^a^ [85.70; 99.48]	97.9 CI [88.9; 99.95]	100.0 CI [92.60; 100.00]
6 months	50	70.0 CI [55.39; 82.14]	88.0 CI [75.69; 95.47]	92.0 CI [80.77; 97.78]
p-level^b^		0.0008*	0.0585	0.0465*

^a^CI: 95% confidence interval

^b^Comparison of the sensitivity 4–10 weeks versus 6 months post infection by the chi-squared test. Significant p-values are labeled with an asterisk

The difference in sensitivity of the tests at 4-10 weeks after infection was statistically insignificant, but the RBD- and NP/RBD-based tests were more sensitive than the NP-based ELISA with the sera from 6 months after infection (p = 0.0413 and 0.0002). The NP/RBD-based ELISA showed a higher percentage of positive results compared with the RBD-based antibody assay, but this was not statistically different (Table 3). Five sera that were positive in the NP ELISA and negative in the RBD test were positive in the NP/RBD-based ELISA (Supplementary Table 1).

**Table 3:** Comparison of the sensitivity of the NP, RBD, and NP/RBD antibody ELISA

	Time post infection					
	> 4 weeks		4–10 weeks		6 months	
ELISA	RBD^a^	NP/RBD	RBD	NP/RBD	RBD	NP/RBD
NP	0.049^b^*	0.0009*	1	0.5	0.0413*	0.0002*
RBD	x	0.687	x	1	x	0.453

^a^*RBD*, receptor-binding domain; *NP*, nucleoprotein

^b^p-values. Sensitivity values from Table 1 were compared with the exact binomial test. An asterisk indicates statistical significance at a significance level of 0.05

## Discussion

Most commercial SARS CoV-2 antibody tests measure the antibody response against either the viral NP or portions of the spike glycoprotein. Some tests measure antibodies against both NP and the spike protein [9, 13, 15, 20–22]. We investigated if simultaneous testing with the NP and RBD improves the diagnostic sensitivity of SARS CoV-2 antibody testing compared with testing for antibodies against the NP and RBD alone.

The diagnostic sensitivity and specificity of the ELISAs were in the same range as commercial NP- and spike glycoprotein–based SARS CoV-2 ELISA tests [18, 23–27]. The AUROC of the NP-based test was significantly smaller than those of the RBD- and NP/RBD-based ELISAs indicating that the NP-based ELISA has lower discriminatory potency. Among the other two assays, the NP/RBD-based test recognized more sera than the RBD-based ELISA. Several sera that were negative in the RBD-based ELISA were positive in both the NP-based and the NP/RBD-based ELISAs. However, the risk of false positive results also increased. With the samples examined, the AUROC and the diagnostic sensitivity of the combined antibody test were slightly higher than those of the RBD-based assay, but this was not statistically significant. The results are similar to those recently obtained with a Luminex-based SARS CoV-2 antibody test that simultaneously measured antibodies against NP, RBD, and other spike protein fractions [22].

We also evaluated the percentage of positive results with sera from different time points. The three ELISAs showed more positive results with sera obtained at 4–10 weeks after infection. In that time period, the NP-, RBD- and NP/RBD-based IgG ELISAs showed comparable proportions of positive sera. When tested with sera obtained 6 months after infection, the RBD- and NP/RBD-based tests detected more sera than the NP-based ELISA. The lower rate of positive results is most likely due to the slightly shorter half-life of NP-specific antibodies in the serum compared to spike glycoprotein–specific antibodies [28–30].

The lower limits of detection of the NP- and RBD-based ELISAs were approximately 30 and 10 IU/ml (First WHO International Standard for anti-SARS-CoV-2 immunoglobulin, NIBSC code 20/136, data not shown). The analytical sensitivity remains unaffected by the antibody concentration in the sera. The loss of diagnostic sensitivity shown here is due to the decline of SARS CoV-2 antibodies after infection [28–30].

The main technical limitation of the ELISAs is the occurrence of false positive sera and plasma. Sera that were false positive in the NP- or the RBD-based ELISA reacted with the NP/RBD-based ELISA. A similar observation was previously made with a Luminex-based antibody test [22]. Thus, although the sensitivity of the ELISA with the two proteins increased, the specificity declined concomitantly. Interestingly, the false positive sera were negative when tested in the nucleoprotein-based Roche Elecsys and the RBD-based Siemens Atellica IM COV2T antibody bridging assays (data not shown). This suggests that further improvement of the ELISA technique may reduce the frequency of false positive reactions.

Most vaccines against SARS CoV-2 induce immune responses against the spike glycoprotein but not the nucleoprotein. Therefore, tests that detect antibodies against the viral NP do not measure vaccine-induced antibodies. On the other hand, NP-specific antibodies are a sign of previous infection. Ideally, SARS CoV-2 antibody tests provide information to differentiate between previous infection and immunization.

In summary, the SARS CoV-2 RBD-specific antibody ELISA was diagnostically more sensitive than the NP-specific ELISA. Simultaneous measurement of NP- and RBD-specific antibodies further increased the percentage of positive sera, but this was not statistically significant due to small sample size. The ELISAs showed fewer positive results with sera from 6 months after infection compared with sera from early time points. The loss of diagnostic sensitivity was most pronounced with the nucleoprotein-specific assay. This suggests that assays using the RBD or NP/RBD are better for serosurveillance beyond 6 months after infection.

## Supplementary Information

ESM 1 (DOCX 6672 kb)

## Data Availability

Original data will be made available upon request.
