# Supplementary material for: Performance of a SARS CoV-2 antibody ELISA based on simultaneous measurement of antibodies against the viral nucleoprotein and receptor-binding domain
Source: Eur J Clin Microbiol Infect Dis. 2021 Jun 4;40(12):2645–9. doi: 10.1007/s10096-021-04284-5 (PMC8175097; doi:10.1007/s10096-021-04284-5)
Supplement: Supplementary file 1 — (DOCX 6672 kb) [file 10096_2021_4284_MOESM1_ESM.docx]

# **Performance of a SARS CoV-2 antibody ELISA based on simultaneous measurement of antibodies against the viral nucleoprotein and receptor-binding domain**

Nina Reiners^1^^[[1]](#footnote-1)^, Carolin Schnurra^1^, Henning Trawinski^2^, Judith Kannenberg^1^, Thomas Hermsdorf^3^, Andrea Aebischer^4^, Torsten Schöneberg^3^, Sven Reiche^4^, Christian Jassoy^1^

**Appendix A. Supplementary Material**

**S1: Production of recombinant proteins**

# Recombinant nucleoprotein: The gene sequence of the nucleoprotein of the SARS-CoV-2/human/AUS/VIC01/2020 isolate (GenBank sequence number MT007544.1. **Coly et al. MJA 2020**) was synthesized (GeneArt. Thermo Scientific). cloned into the plasmid pMal-c5e plasmid (NEB) and expressed as a maltose-binding protein (MBP) fusion protein in E. coli strain BL21 DE3. Transformed *E. coli* cells were cultivated overnight in 200 ml LB medium with 10 µ/ml ampicillin in a shaking incubator. Four liters of LB medium with ampicillin were inoculated with the overnight culture (OD600 0.2). Cells were grown at 37 °C in a shaking incubator to OD_600 nm_ of 0.6 to 0.8 followed by addition of IPTG (final concentration 400 µM). After 150 min of incubation. bacteria were harvested by centrifugation at 10.000 g and 4 °C. The pellet (6-8 g) was resuspended at 4 °C in 40 ml 20 mM Tris-Cl. 1 mM EDTA. 150 mM NaCl pH 7.4 (binding buffer) containing 7 mg lysozyme and a protease inhibitor cocktail (Roche). The solution was stirred for 30 min at 4°C. The *E. coli* cells were lysed in a prechilled French Press chamber (Aminco SLM) at 1200 psig. The lysate was centrifuged at 30.000 g and 4 °C for 30 min. To precipitate the DNA. protamine sulphate was stepwise added to the supernatant (final concentration of 0.2 %) and stirred for 5 min at 4 °C. The suspension was centrifuged at 30.000 g and 4 °C for 30 min. The supernatant was immediately applied to an MBP-TRAP^TM^ HP column (5 ml. GE Healthcare) and purified with an Äkta pure protein purification system (GE Healthcare). Protein was eluted with 10 mM maltose in binding buffer. Eluates were dialysed against the binding buffer and subjected to SDS polyacrylamide gel electrophoresis for quality analysis (Suppl. Fig. 1).

Recombinant receptor binding domain (RBD): The SARS-CoV2 RBD-SD1 domain (amino acids 319 – 519 of the SARS-CoV2 Spike ectodomain. [QHD43416]) was amplified from a codon-optimized synthetic gene (GeneArt. Thermo Scientific). The construct was cloned in the expression vector pEXPR103 (iba lifesciences) in frame with an N-terminal modified mouse Ig kappa light chain signal peptide and a c-terminal double strep tag. For recombinant protein expression Expi293 cells were grown in Expi293 expression medium (Thermo Scientific) and polycarbonate Erlenmeyer flasks (Corning) at 37 °C. 8% CO2. 125 rpm. Transfection was performed using the ExpiFectamine293 transfection kit (Thermo Scientific) according to the manufacturer´s instructions. Five days after transfection the cells were harvested by centrifugation at 6.000 x g for 20 min at 4 °C. Biotin was blocked by addition of BioLock (iba lifesciences) as recommended and the supernatant was purified using Strep-Tactin XT Superflow high capacity resin (iba lifesciences) according to the protocol of the manufacturer. The proteins were eluted with 50 mM Biotin (in 100 mM Tris-HCl. 150 mM NaCl. 1 mM EDTA; pH 8.0) and stored at -80 °C until further use.

**Supplementary Figure 1**


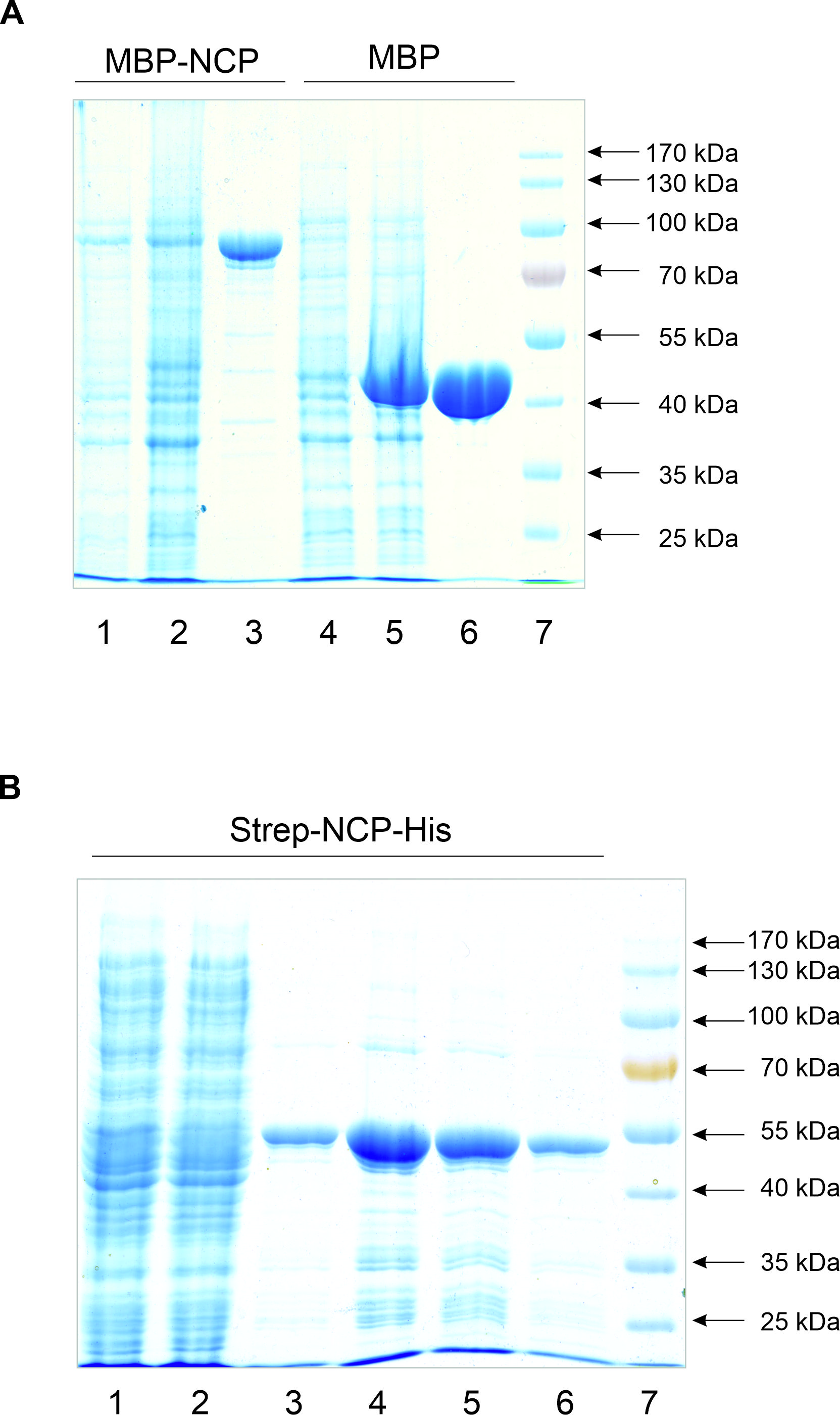

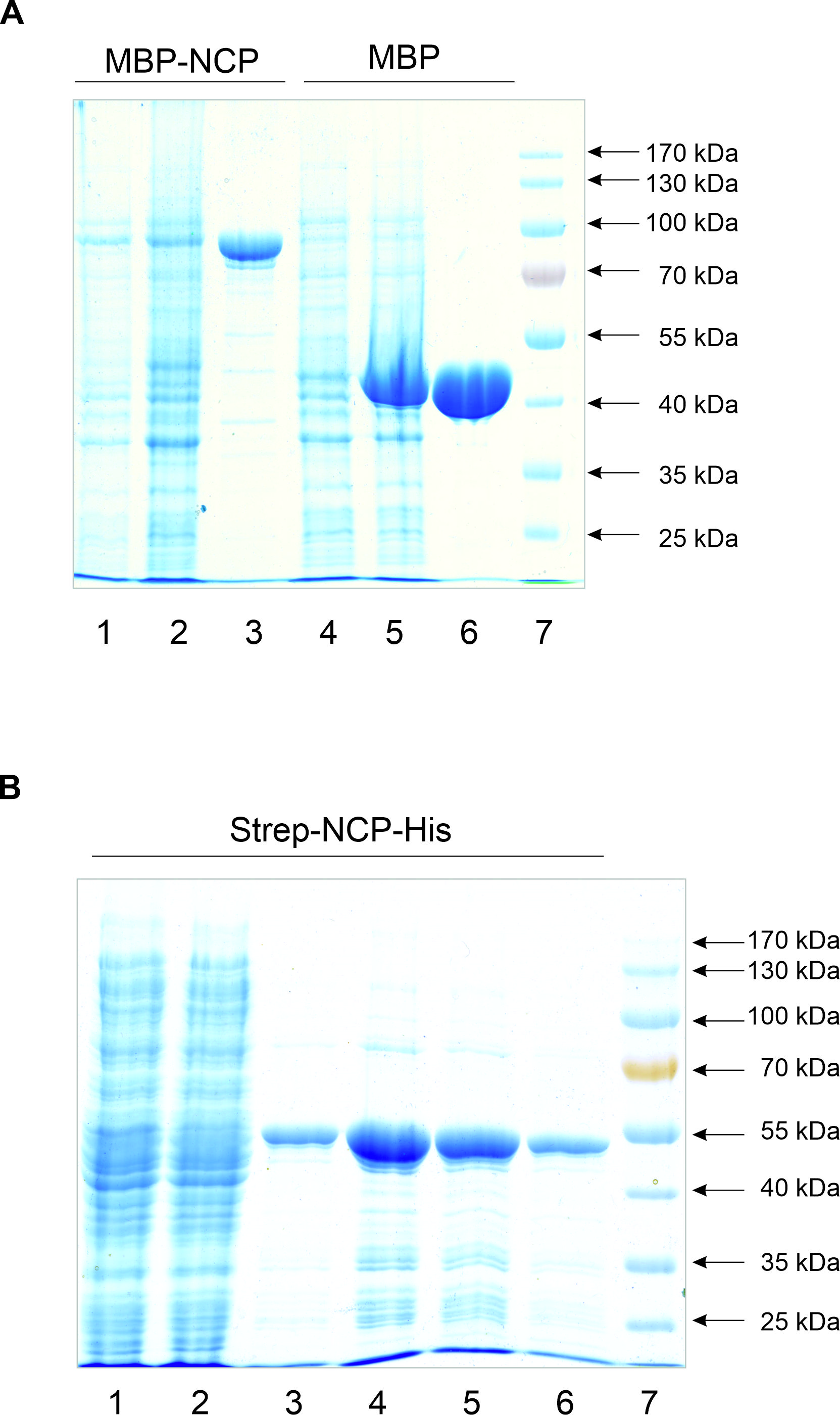


NP-MBP

1 2 3 4

**Fig. 1** SDS-PAGE analysis of lysate of transformed E. coli before induction (lane 1) and after 2.5 h induction with IPTG (lane 2) and eluted protein from maltose binding protein affinity column (lane 3). Theoretical molecular weight is 88.5 kDa for nucleoprotein maltose binding protein fusion protein (NP-MBP). Lane 4: Molecular weight marker PageRuler prestained protein ladder (Themo scientific).

**S2: Antibody ELISA protocol**

High-binding microtiter plates (Greiner BioOne) were coated with 1 μg/ml NP-MBP or RBD-TwinStrep-Tag fusion protein or with 1 µg/ml RBD and 0.5 μg/ml NP in phosphate-buffered saline (PBS), pH 7.4. Plates were incubated overnight at 4 °C or for 60 minutes at 37 °C. All further steps were performed at room temperature. Plates were washed with deionized water and washing buffer (PBS, 0.05% Tween-20) and blocked for 20 minutes with blocking solution (PBS containing 0.05% tween-20 and 5% milk powder). The sera were diluted 1:100 in blocking solution and incubated for 1 hour. The plates were washed and a rabbit-anti-human IgG conjugated with HRP (Art. No. P0214, Dako), diluted 1:100.000 for the NP-based test and 1:50.000 for the RBD and the NP/RBD-based tests) was added for 1 hour. The plates were washed and 3.3′,5.5′-Tetramethylbenzidine (Scytek TMB Soluble Reagent, Dianova GmbH or SeramunBlau slow 2/85, Seramun Diagnostica GmbH) was utilized as the substrate. The enzymatic reaction was stopped after 15 minutes by addition of 1 N sulphuric acid. The optical density (OD) was measured with a photometer at a wavelength of 450 nm and a reference wavelength of 570 nm. Control experiments with E. coli maltose binding protein prepared in a similar fashion as the NP-MBP fusion protein showed optical densities similar to blank control wells.

**Supplementary Table 1: Antibody ELISA results with sera from SARS CoV-2 RT-PCR positive subjects**

1. **4-10 weeks post infection**

|  | **Antibody ELISA units (Cutoff value)** | | |
| --- | --- | --- | --- |
| **Serum** | **NP**  **(>0.760)** | **RBD (>0.948)** | **NP/ RBD (>0.957)** |
| **CoV045-1** | 6.52 | 15.33 | 12.33 |
| **CoV002-1** | 2.99 | 8.91 | 6.72 |
| **CoV003-1** | 4.76 | 9.29 | 11.44 |
| **CoV004-1** | 6.71 | 11.14 | 10.48 |
| **CoV005-1** | 1.68 | 5.73 | 5.31 |
| **CoV006-1** | 4.29 | 9.94 | 10.70 |
| **CoV012-1** | 2.39 | 1.38 | 2.11 |
| **CoV014-1** | 2.74 | 4.51 | 5.39 |
| **CoV015-1** | 1.94 | 3.31 | 4.37 |
| **CoV018-1** | 1.60 | 2.46 | 3.15 |
| **CoV019-1** | 2.26 | 4.51 | 6.77 |
| **CoV021-1** | 3.58 | 13.29 | 10.09 |
| **CoV022-1** | 5.17 | 6.66 | 9.03 |
| **CoV023-1** | 2.85 | 7.33 | 6.83 |
| **CoV024-1** | 1.72 | 9.45 | 9.06 |
| **CoV026-1** | 4.12 | 17.21 | 12.63 |
| **CoV027-1** | 4.97 | 3.23 | 6.18 |
| **CoV028-1** | 3.44 | 4.53 | 6.94 |
| **CoV029-1** | 5.09 | 4.56 | 8.31 |
| **CoV030-1** | 4.29 | 18.40 | 13.95 |
| **CoV031-1** | 2.71 | 4.02 | 5.27 |
| **CoV032-1** | 2.75 | 7.69 | 8.80 |
| **CoV033-1** | 1.17 | 1.58 | 2.42 |
| **CoV038-1** | 2.10 | 4.02 | 4.24 |
| **CoV039-1** | 2.12 | 7.02 | 6.33 |
| **CoV043-1** | 1.70 | 2.87 | 3.98 |
| **CV220/002-2** | 1.89 | 3.46 | 3.05 |
| **CV220/008-2** | 3.24 | 5.92 | 6.29 |
| **CV220/012-2** | 3.14 | 8.77 | 5.98 |
| **CV220/013-2** | 3.76 | 7.48 | 5.94 |
| **CV220/001-2** | 6.30 | 13.38 | 8.70 |
| **CV220/006-2** | 4.94 | 12.18 | 11.00 |
| **CV220/010-2** | 6.42 | 22.30 | 14.44 |
| **CV220/011-2** | 3.62 | 9.90 | 6.90 |
| **CV220/024-2** | 2.53 | 4.96 | 4.45 |
| **CV220/026-2** | 1.40 | 2.23 | 3.05 |
| **CV220/035-2** | 5.08 | 7.64 | 5.73 |
| **CV220/039-2** | 2.67 | 1.71 | 3.32 |
| **CoV020-1** | 4.16 | 0.61 | 2.68 |
| **CoV017-1** | 0.95 | 2.49 | 2.31 |
| **CoV040-1** | 0.770 | 1.35 | 1.81 |
| **CoV011-1** | 0.30 | 1.84 | 2.34 |
| **CoV036-1** | 0.773 | 6.79 | 4.26 |
| **CoV044-1** | 0.87 | 1.51 | 1.79 |
| **CV220/003-2** | 0.79 | 1.93 | 1.90 |
| **CV220/022-2** | 0.82 | 1.95 | 1.84 |
| **CV220/021-2** | 0.86 | 2.85 | 1.29 |
| **CV220/027-2** | 0.71 | 6.15 | 3.59 |

1. **6 months after infection**

|  | **Antibody ELISA units (Cutoff value)** | | |
| --- | --- | --- | --- |
| **Serum** | **NP**  **(>0.760)** | **RBD (>0.948)** | **NP/ RBD (>0.957)** |
| **CoV001-2** | 1.18 | 4.7 | 2.56 |
| **CoV002-2** | 1.46 | 5.7 | 4.73 |
| **CoV003-2** | 3.39 | 6.0 | 6.55 |
| **CoV004-2** | 8.01 | 8.6 | 14.23 |
| **CoV006-2** | 1.87 | 3.5 | 3.59 |
| **CoV013-2** | 3.35 | 16.2 | 9.55 |
| **CoV014-2** | 1.00 | 4.7 | 3.74 |
| **CoV021-2** | 1.32 | 7.4 | 3.79 |
| **CoV022-2** | 3.66 | 3.2 | 5.51 |
| **CoV023-2** | 1.77 | 4.1 | 3.62 |
| **CoV026-2** | 2.60 | 11.7 | 7.93 |
| **CoV027-2** | 1.88 | 1.6 | 3.29 |
| **CoV028-2** | 1.71 | 3.6 | 3.03 |
| **CoV029-2** | 3.09 | 12.6 | 9.55 |
| **CoV030-2** | 4.87 | 4.0 | 7.28 |
| **CoV031-2** | 1.21 | 2.5 | 2.91 |
| **CoV032-2** | 1.22 | 4.6 | 3.78 |
| **CoV035-2** | 2.02 | 4.2 | 4.58 |
| **CoV039-2** | 2.31 | 5.6 | 5.97 |
| **CoV045-2** | 7.39 | 13.4 | 9.73 |
| **CoV046-1** | 1.21 | 1.2 | 1.92 |
| **CV220/001-3** | 3.69 | 4.8 | 5.11 |
| **CV220/006-3** | 3.73 | 4.4 | 5.77 |
| **CV220/008-3** | 1.54 | 2.1 | 2.45 |
| **CV220/010-4** | 4.71 | 8.8 | 7.56 |
| **CV220/011-5** | 1.25 | 5.5 | 3.36 |
| **CV220/024-3** | 1.24 | 6.5 | 4.72 |
| **CV220/035-3** | 4.00 | 4.9 | 7.20 |
| **CoV005-2** | 0.32 | 8.5 | 4.21 |
| **CoV015-2** | 0.82 | 2.1 | 2.00 |
| **CoV017-2** | 0.64 | 2.5 | 1.79 |
| **CoV018-2** | 0.60 | 1.2 | 1.70 |
| **CoV019-2** | 0.75 | 3.0 | 2.37 |
| **CoV024-2** | 0.54 | 8.3 | 4.09 |
| **CoV036-2** | 0.37 | 2.0 | 1.58 |
| **CoV038-2** | 0.56 | 4.5 | 3.39 |
| **CoV043-2** | 0.93 | 1.7 | 1.46 |
| **CV220/002-3** | 0.43 | 1.4 | 1.21 |
| **CV220/013-3** | 0.69 | 5.8 | 1.83 |
| **CV220/021-3** | 0.74 | 2.0 | 1.47 |
| **CV220/022-3** | 0.57 | 0.97 | 1.15 |
| **CV220/026-3** | 0.92 | 1.4 | 1.66 |
| **CV220/039-3** | 0.85 | 0.87 | 1.54 |
| **CoV012-2** | 1.52 | 0.7 | 1.77 |
| **CoV020-2** | 1.87 | 0.4 | 2.32 |
| **CoV033-2** | 0.83 | 0.7 | 1.01 |
| **CoV044-2** | 0.48 | 0.97 | 0.83 |
| **CV220/003-3** | 0.28 | 0.7 | 0.58 |
| **CoV011-2** | 0.29 | 3.0 | 0.90 |
| **CoV040-2** | 0.25 | 0.92 | 0.47 |

1. N. Reiners and C. Schnurra contributed equally to the study [↑](#footnote-ref-1)
